# P-2064. COVID-19 Vaccination-infection Status and Immunological Profile: Identification of High-Risk Population for Targeted Booster Immunization In India

**DOI:** 10.1093/ofid/ofae631.2220

**Published:** 2025-01-29

**Authors:** Ankit Mittal, Deepika Gujjarlapudi, Vidyavathi devi Gajapathi Raju, Sadhana Yelamanchili Veturi, Rupjyoti Talukdar, Rupa Banerjee, Nitin Jagtap, Sannapaneni Krishnaiah, Namburu Veeraiah, Duvvur Nageshwar Reddy

**Affiliations:** AIG Hospitals, Hyderabad, Uttar Pradesh, India; AIG Hospitals, Hyderabad, Uttar Pradesh, India; AIG Hospitals, Hyderabad, Uttar Pradesh, India; AIG Hospitals, Hyderabad, Uttar Pradesh, India; AIG Hospitals, Hyderabad, Uttar Pradesh, India; AIG Hospitals, Hyderabad, Uttar Pradesh, India; AIG Hospitals, Hyderabad, Uttar Pradesh, India; AIG Hospitals, Hyderabad, Uttar Pradesh, India; AIG Hospitals, Hyderabad, Uttar Pradesh, India; AIG Hospitals, Hyderabad, Uttar Pradesh, India

## Abstract

**Background:**

The immunological profiles and predictors of immune response in people with hybrid immunity, resulting from a combination of natural infection and vaccination, particularly in regions where non-mRNA vaccines were administered, remain less understood.

**Figure:**
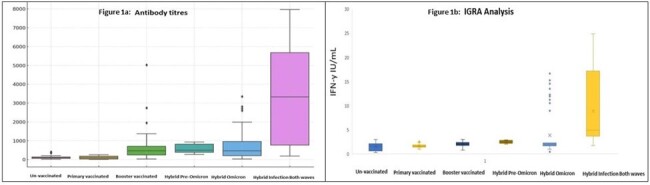

**Methods:**

This was a cross-sectional study done at AIG Hospitals, Hyderabad, India. Consecutive participants were enrolled, and sampled for immunological testing. The study population was divided into six cohorts based on their infection and vaccination status. Immunological assays were performed to measure anti-spike protein and interferon-γ release assay. Hospitalization rates were compared in all groups. Chi-square test was used to compare categorical variables, and the Mann-Whitney U test was used for comparing medians. Post-hoc Dunn test was used for pairwise comparison. Bonferroni adjustment for p-value was performed for multiple pairwise comparisons. Linear regression model was used appropriately to find predictors of antibody titres.

**Figure:**
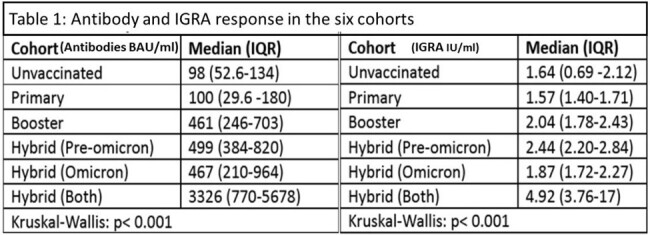

**Results:**

A total of 1040 patients were enrolled in the study. The population was divided into six cohorts: Unvaccinated, Primary, Booster, Hybrid (Pre-Omicron only), Hybrid (Omicron only), and Hybrid (Both). Antibody titres and IGRA levels were found to be statistically higher in the Hybrid (both infection) group (Table 1 and Figures 1a & 1b). The linear regression model showed factors predicting antibody response (Table 2). Age and hypertension were independent (negative) predictors of antibody levels.

During the follow-up (median 30.7 [26.6 – 34.4 months]) of 1008 patients, 770 (76.4%) had a documented COVID infection with Omicron. Hospitalization was evaluated in three groups: Hybrid, vaccination only and unvaccinated. The incidence was significantly lower in the Hybrid group; log-rank test: p < 0.001. (Figure 2)

**Figure:**
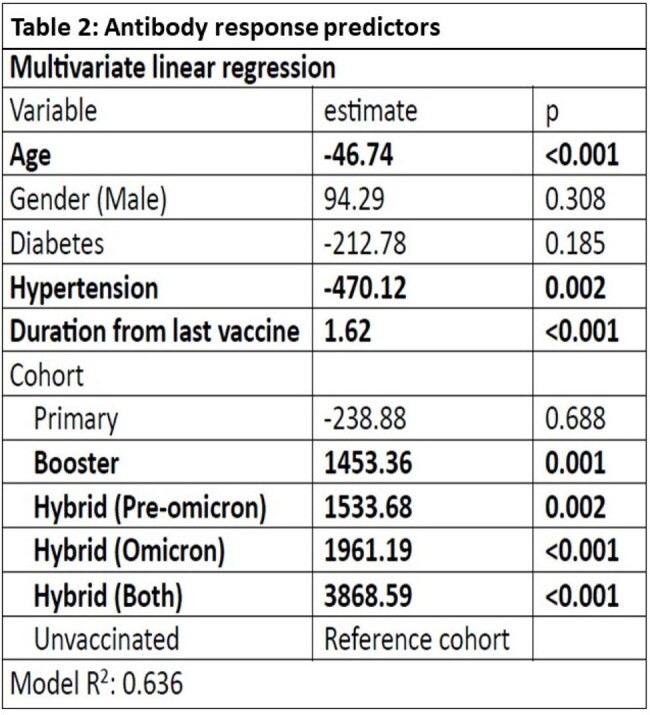

**Conclusion:**

The prevalence of hybrid immunity was high in the study population. Individuals above 60 years old and with comorbidities are more likely to have lower antibody titers. Immunized patients were protected against hospitalization. There is a need for prioritized immune-boosting efforts in high-risk individuals.

**Figure:**
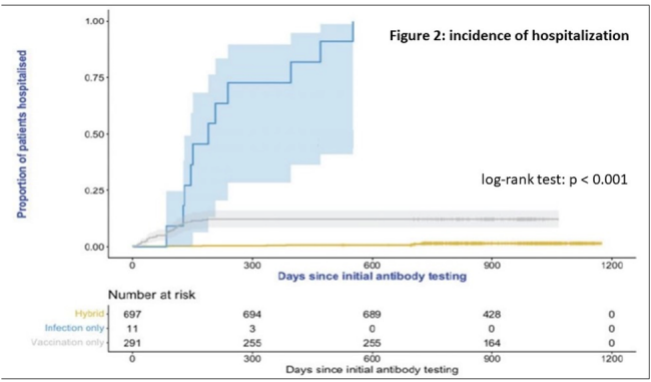

**Disclosures:**

All Authors: No reported disclosures

